# Genetical and epigenetical profiling identifies two subgroups of pineal parenchymal tumors of intermediate differentiation (PPTID) with distinct molecular, histological and clinical characteristics

**DOI:** 10.1007/s00401-023-02638-1

**Published:** 2023-09-30

**Authors:** Ramin Rahmanzade, Elke Pfaff, Rouzbeh Banan, Philipp Sievers, Abigail K. Suwala, Felix Hinz, Henri Bogumil, Asan Cherkezov, Aras Fuat Kaan, Daniel Schrimpf, Dennis Friedel, Kirsten Göbel, Felix Keller, Xavier Saenz-Sardà, Alexander Lossos, Martin Sill, Olaf Witt, Oliver W. Sakowitz, Andrey Korshunov, David E. Reuss, Nima Etminan, Andreas Unterberg, Miriam Ratliff, Christel Herold-Mende, Wolfgang Wick, Stefan M. Pfister, Andreas von Deimling, David T. W. Jones, Felix Sahm

**Affiliations:** 1 Department of Neuropathology, Institute of Pathology, University Hospital Heidelberg, Heidelberg, Germany; 2 Clinical Cooperation Unit Neuropathology, German Consortium for Translational Cancer Research (DKTK), German Cancer Research Center (DKFZ), Heidelberg, Germany; 3 Hopp Children’s Cancer Center Heidelberg (KiTZ), Heidelberg, Germany; 4 Department of Pediatric Oncology, Hematology, Immunology and Pulmonology, University Hospital Heidelberg, Heidelberg, Germany; 5 Division of Pediatric Glioma Research, German Cancer Research Center (DKFZ), Heidelberg, Germany; 6 Division of Experimental Neurosurgery, Department of Neurosurgery, University Hospital Heidelberg, Heidelberg, Germany; 7 Department of Neurosurgery, University Hospital Mannheim, University of Heidelberg, Mannheim, Germany; 8 Clinical Cooperation Unit Neurooncology, German Cancer Consortium (DKTK), German Cancer Research Center (DKFZ), 69120 Heidelberg, Germany; 9 Division of Pathology, Department of Clinical Sciences Lund, Lund University, Lund, Sweden; 10 Departments of Oncology and Neurology, Leslie and Michael Gaffin Center for Neuro-Oncology, Hadassah-Hebrew University Medical Center, Jerusalem, Israel; 11 Clinical Cooperation Unit Pediatric Oncology, German Consortium for Translational Cancer Research (DKTK), German Cancer Research Center (DKFZ), Heidelberg, Germany; 12 Neurosurgery Center Ludwigsburg-Heilbronn, RKH Klinikum Ludwigsburg, Ludwigsburg, Germany; 13 Department of Neurosurgery, University Hospital of Heidelberg, Heidelberg, Germany; 14 Clinical Cooperation Unit Neurooncology, German Consortium for Translational Cancer Research (DKTK), German Cancer Research Center (DKFZ), Heidelberg, Germany; 15 Department of Neurology and Neurooncology Program, National Center for Tumor Diseases, Heidelberg University Hospital, Heidelberg, Germany; 16 Department of Neuropathology, University Hospital Heidelberg, Heidelberg, Germany; 17 Clinical Cooperation Unit Neuropathology (B300), German Cancer Consortium (DKTK), German Cancer Research Center (DKFZ), Im Neuenheimer Feld 224, 69120 Heidelberg, Germany

Pineal parenchymal tumors of intermediate differentiation (PPTIDs) are a subset of pineal parenchymal tumors (PPTs) with histological and biological features between well-differentiated pineocytoma and poorly differentiated pineoblastoma [10]. These tumors present diagnostic challenges, and recent molecular profiling has resulted in a substantial rate of reclassification [8, 9]. Previous attempts to stratify PPTIDs into distinct groups based on mitotic count and neurofilament expression, although initially implemented into the 2007 WHO classification, did not show significant relevance in subsequent studies [1–3]. Hence, there is currently no definite grading criteria for PPTIDs in the latest WHO classification [1]. PPTIDs have been classified morphologically into diffuse, lobular, pleomorphic and transitional subtypes (Fig. 1 and Supplementary Figs. 1–4) [1–3]. In addition, they have been epigenetically categorized as PPTID-A and PPTID-B [2]. Small insertions in the *KBTBD4* gene have been suggested to be characteristic for PPTIDs, albeit not present in every case [5, 7]. The prognostic significance of *KBTBD4* insertion is still unclear [7]. Furthermore, the association between morphological and molecular subtypes of PPTIDs, and the prognostic significance of these subtypes remains largely unknown.

**Fig. 1:**
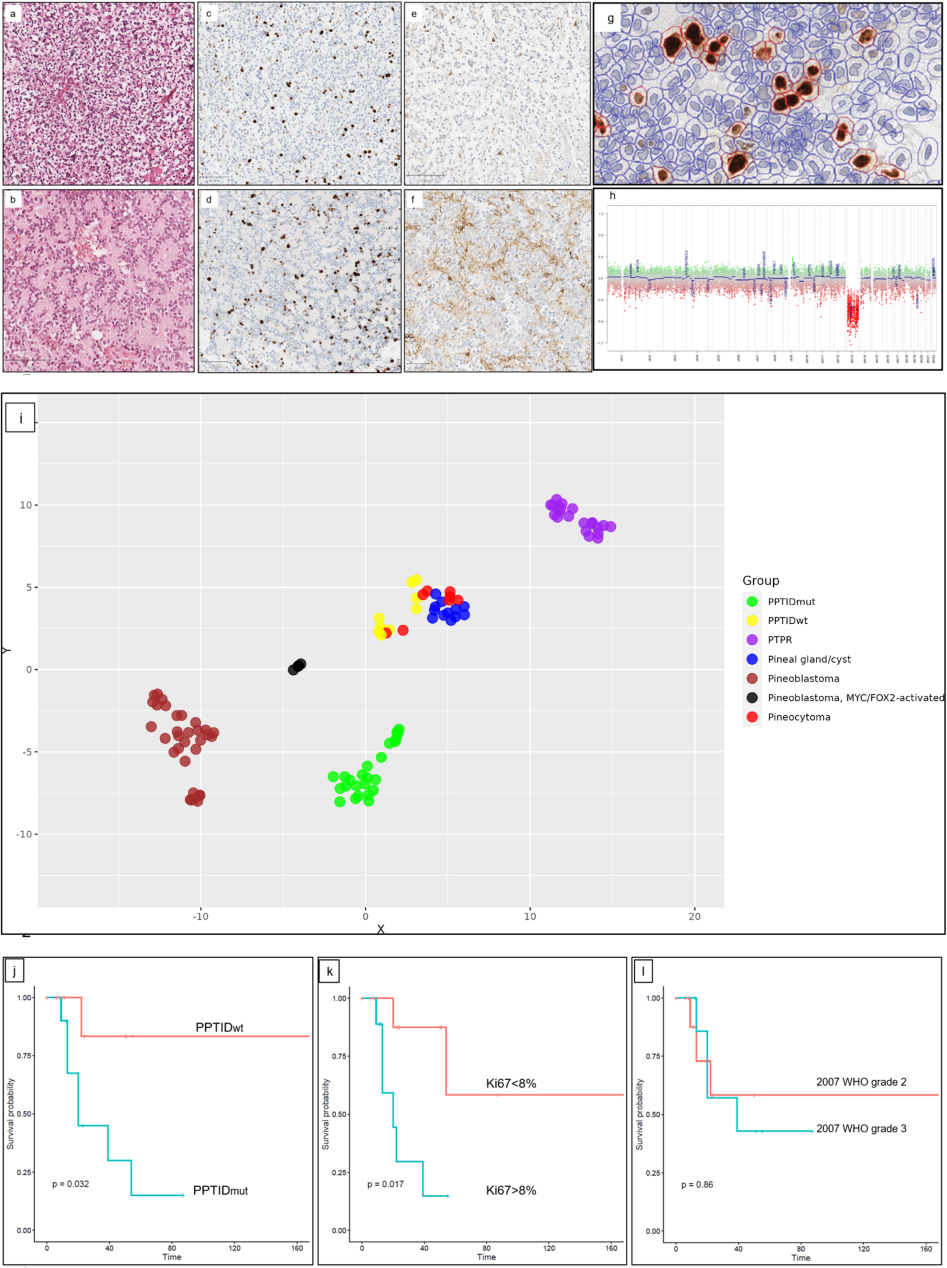
**a**–**h** PPTID_wild_ of transitional subtype depicting areas with diffuse growth pattern and clear-cell morphology (**a**) and, also, rosette-forming areas with typical histology of pineocytoma (**b**). Although both areas showed elevated proliferation index (**c** and **d**, hotspot Ki67: 8.7%), the neurofilament protein was significantly less expressed in diffuse area (**e** and **f**). Hotspot-Ki67 was measured via QuPath in order to reduce the inter-observer variability (**g**). Molecular analyses revealed a wild-type *KBTBD4* gene, loss of chromosome 13q (**h**) and a DNA methylation class distinct from PPTID-A and -B.** i** Unsupervised DNA methylation-based t-SNE showed that PPTID_wt_ are epigenetically more similar to pineocytoma than PPTID_mut_. **j**–**l** Kaplan–Meier curves by log-rank test. *KBTBD4* insertions (*j*, *p* value 0.03) and hotspot Ki67 greater than or equal to 8% (**k**, *p* value 0.02) were significantly associated with worse progression-free survival (PFS). The 2007 WHO grading system (**l**, *p* value 0.8) failed to correlate with the PFS

In an effort to address these challenges, we studied a cohort of 34 PPTID patients. The cases have been diagnosed and included into the study following a thorough review of histological slides and molecular findings of all pineal tumors from our internal database for which the diagnostic slides and DNA material were available (*n* = 110).

All PPTID samples were subjected to comprehensive histological and molecular analyses. The findings were correlated with patients’ survival for 19 patients with available survival data.

The material and methods, as well as the clinical and histological findings, are summarized in the Supplementary data, online resource.

*KBTBD4* insertions were detected in 24 out of 34 PPTIDs. PPTIDs with wild type (PPTID_wt_) and mutant (PPTID_mut_) *KBTBD4* gene showed no significant difference in proliferation index (hotspot Ki67 measured via QuPath software), mitotic count, age and sex distribution. PPTID_mut_ had significantly higher cell density (12,160 vs. 7954 cells/mm^2^; *p* value < 0.001), a smaller cell size (78 vs. 100 µm^2^, *p* value < 0.001), and more frequently showed profound loss of NFP expression compared to PPTID_wt_ (70 vs. 20%, *p* value 0.02). PPTID_mut_ and PPTID_wt_ were mostly of diffuse (62%) and transitional subtypes (70%), respectively.

Genome-wide DNA methylation analysis showed that the 10 PPTID_wt_ clustered separate from the 24 PPTID_mut_, but together with pineal gland/cyst and pineocytoma (Fig. 1i). Loss of chromosome 13q was more common in PPTID_wt_ than PPTID_mut_ (60 vs. 13%, *p* value < 0.01), mostly as the single alteration in the copy-number profiles of PPTID_wt_. Of note, only one of the 8 pineocytoma in our cohort showed loss of chromosome 13q.

There was one radiological tumor recurrence in PPTID_wt_ during follow-up (*n* = 9, mean 44, range 6–169 months), whereas recurrences were documented in 7 out 10 PPTID_mut_ (mean 29, range 9–87 months). The presence of insertions in *KBTBD4*, methylation class PPTID, diffuse morphology subtype and hotspot Ki67 greater than or equal to 8% were significantly associated with a worse progression-free survival (PFS) by log-rank test (Fig. 1j–k and Supplementary Fig. 5). The 2007 WHO grading system showed no correlation with the PFS (*p* value 0.8; Fig. 1l). Insertion in *KBTBD4* was the only significant variable (*p* value 0.04) in the Cox regression model that incorporates *KBTBD4* status, hotspot-ki67, morphology subtype, mitotic count, extent of resection (EOR) and adjuvant therapy. Although prior works are controversial regarding the prognostic significance of EOR and adjuvant therapy [4, 6], they failed to reach the level of significance for association with PFS in our study (Supplementary Table 1 and Supplementary Fig. 6).

In summary, genetic and epigenetic profiling identifies two broader subgroups of PPTIDs with distinct histological features and clinical course: (1) the PPTIDs with *KBTBD4* insertions which have the methylation profile subtypes PPTID-A or PPTID-B and frequently have a small-cell morphology and an unfavourable clinical course and (2) the PPTIDs without *KBTBD4* insertions, herein suggested to be called PPTID-C, which have a methylation profile most similar to pineocytoma and frequently show a large-cell morphology, loss of chromosome 13q, and a favourable clinical course. The incorporation of this subgrouping in grading of PPTIDs may improve risk stratification.

### Supplementary Information

Below is the link to the electronic supplementary material.

Supplementary file1 (DOCX 7449 KB)

Supplementary file2 (DOCX 22 KB)

## Data Availability

The data, summarized in Supplementary file 1, are available at Department of Neuropathology, University Hospital Heidelberg.
