# Supplementary material for: Genetical and epigenetical profiling identifies two subgroups of pineal parenchymal tumors of intermediate differentiation (PPTID) with distinct molecular, histological and clinical characteristics
Source: Acta Neuropathol. 2023 Sep 30;146(6):853–6. doi: 10.1007/s00401-023-02638-1 (PMC10627898; doi:10.1007/s00401-023-02638-1)
Supplement: Supplementary file 1 — Supplementary file1 (DOCX 7449 KB) [file 401_2023_2638_MOESM1_ESM.docx]

**Supplementary Appendix**

Supplement to: Rahmanzade R, Pfaff E, et al. **Genetical and epigenetical profiling identifies two subgroups of Pineal parenchymal tumors of intermediate differentiation (PPTID) with distinct molecular, histological and clinical characteristics**

**Table of contents**

Supplementary Figures ..................................................................................2-7

Supplementary Tables....................................................................................8

Corresponding authors:

**Felix Sahm, MD**

Department of Neuropathology

University Hospital Heidelberg

and

Clinical Cooperation Unit Neuropathology (B300)

German Cancer Research Center (DKFZ)

Im Neuenheimer Feld 224

69120 Heidelberg, Germany

Fon: +49-6221 56-37886; Fax: +49-6221 56-4566

Felix.sahm@med.uni-heidelberg.de


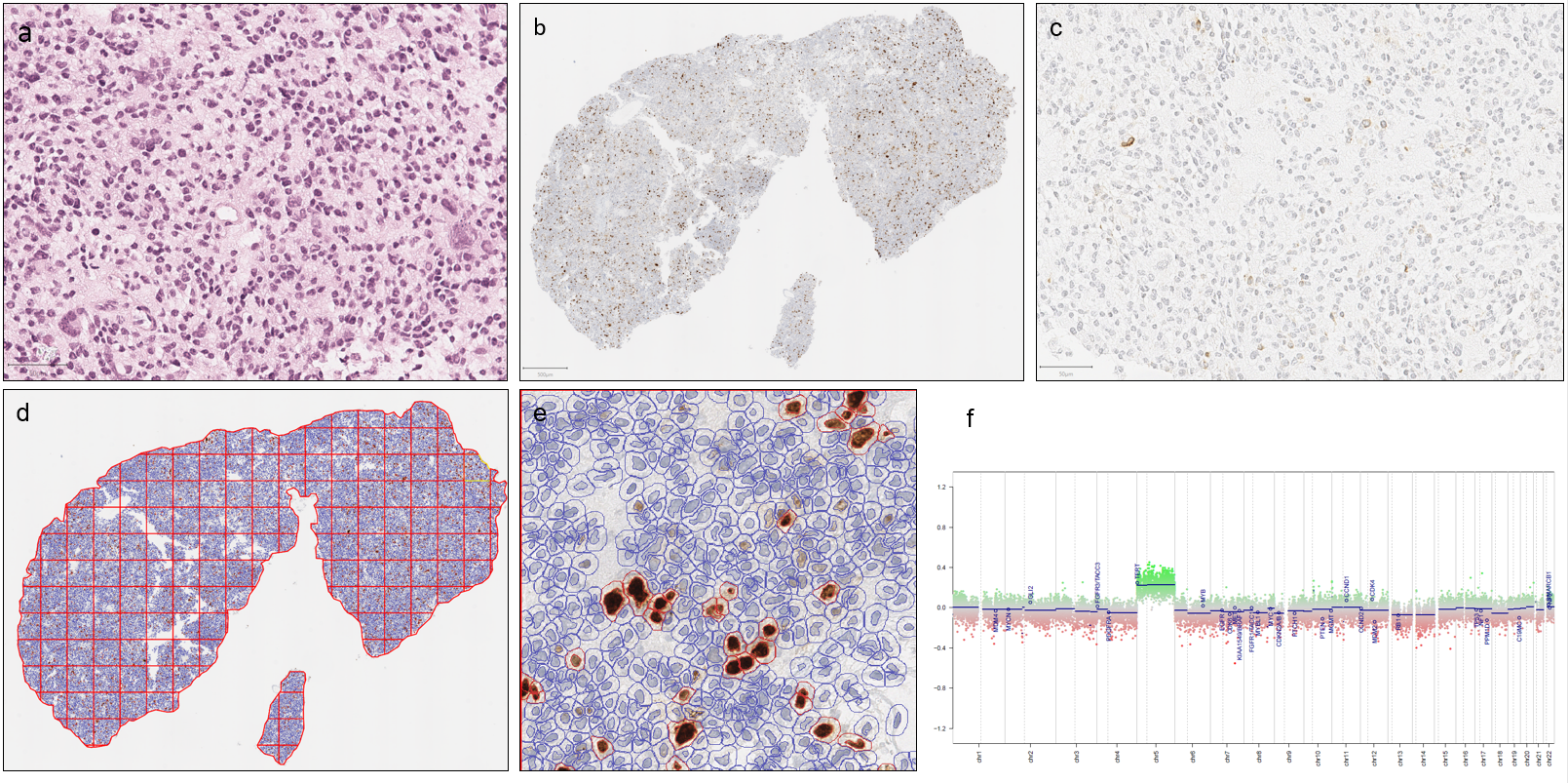


**Supp. Fig. 1**

PPTID_wt_ of pleomorphic subtype with multinucleated giant cells (a) increased proliferarive activity (b) and expression of neurofilament in less than 10% of tumor cells (c). The grid size of 500µm (d) and a threshold of 0.5 for staining intensity (e) was applied to increase the specificity of Ki67-positive nuclei detection and hotspot Ki67 measurement. The avarage of the top five scores was reported as hotspot Ki67. As shown in (e), the weakly stained neuclei were omitted and the analysis resulted a hotspot Ki67 of 9.7%. Molecular analyses revealed a wild-type *KBTBD4* gene, broad gain of chromosome 5 (f) and a DNA methylation class distinct from PPTID-A and -B. There was no evidence of the tumor recurrence in the last follow up about 4.5 years after surgery.


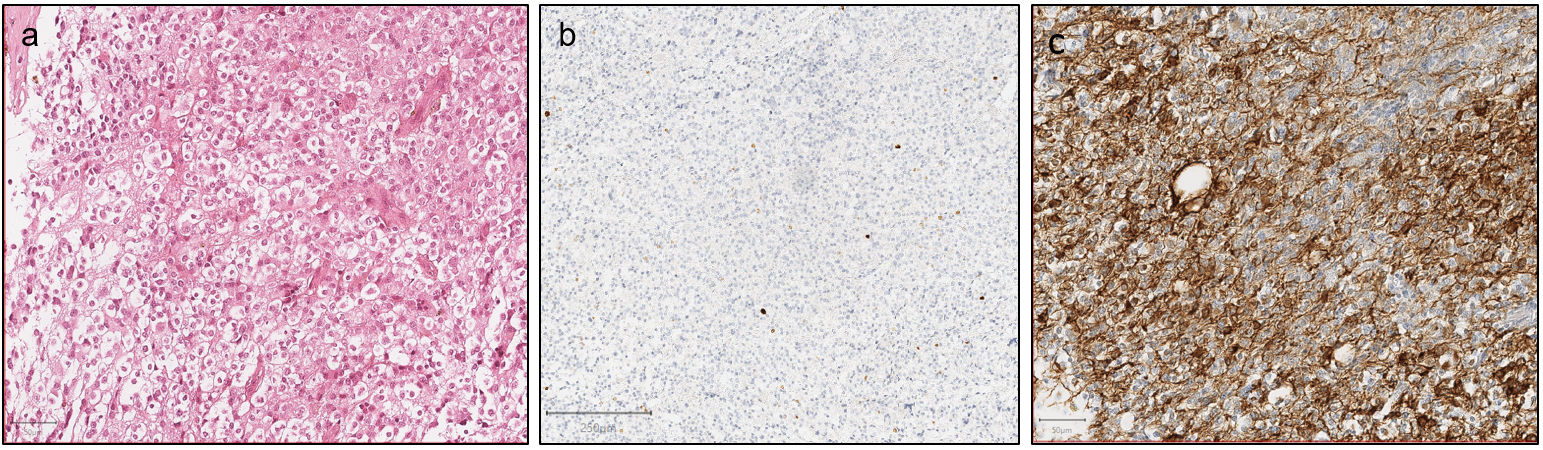


**Supp. Fig. 2**

Pineal parechymal tumor predominantly showing a diffuse growth pattern and clear-cell morphology (a). Immunhistochemical examiniations revealed diffuse staining for synaptophysin (not shown) and neurofilament protein (c). The tumor showed no mitotic figures and a low proliferation index (b; hotspot Ki67 of 1.1%). Molecular analyses revealed a wild-type *KBTBD4* gene and DNA methylation class distinct from PPTID-A and -B. While not showing the typical pineocytomatous rosettes, the tumor was diagnosed as pineocytoma, WHO grade 1 according to the 2021 WHO classification of brain tumors.


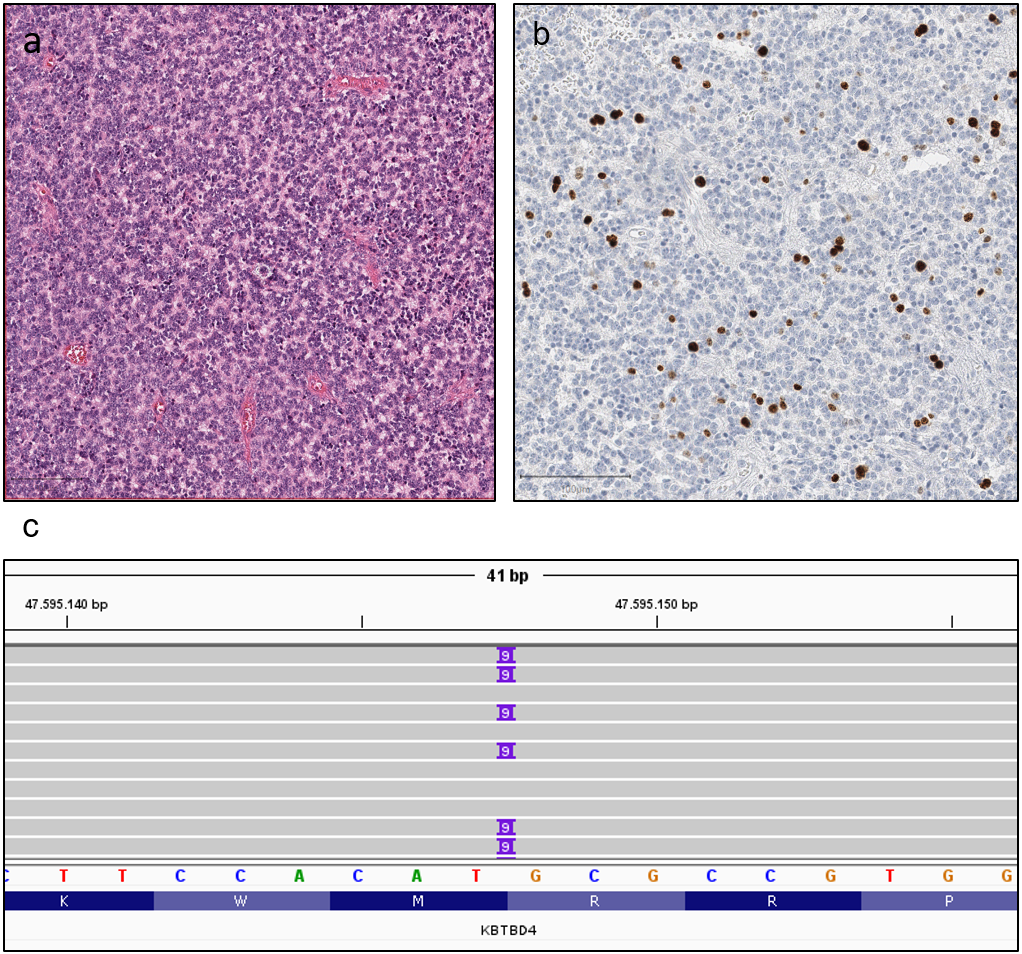


**Supp. Fig. 3**

PPTID_mut_ of diffuse subtype and small cell morphology (mean tumor cell area: 84µm^2^) showing frequent small Homer-Wright rosettes (a) and elevated proliferation index (b; hotspot Ki67: 6.1%). Next-Generation sequencing revealed insertions of 9 bases at codon 313-314 of *KBTBD4* gene and DNA methylation analysis showed methylation class of PPTID-B (c). There was evidence of a tumor recurrence 54 months after surgery.


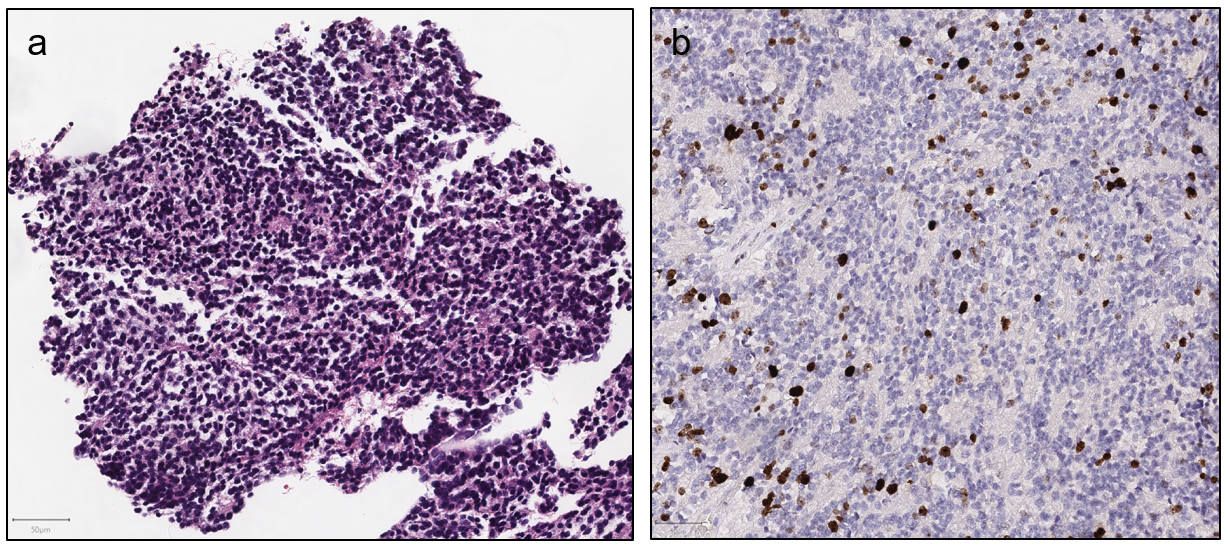


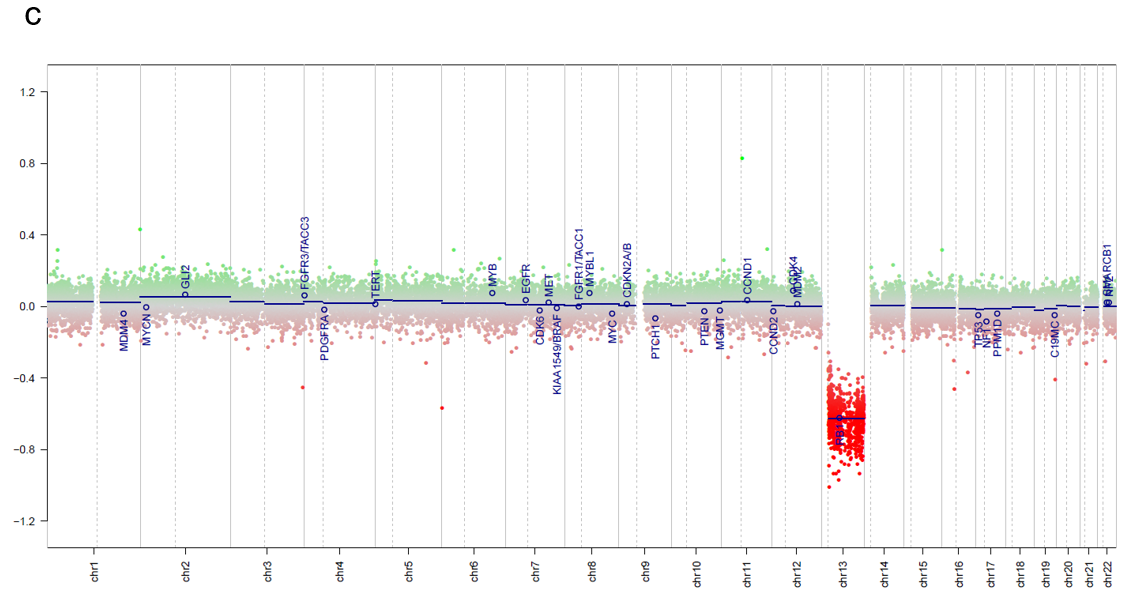


**Supp. Fig. 4**

PPTID_wt_ of transitional subtype and small cell morphology (71 µm^2^) and high cell density (13045 cells/ 1mm^2^) (a). Immunhistochemical examinations showed elevated proliferative activity (hotspot Ki67: 8.3%) (b). The irregular pineocytomatous rosettes are visible in (a) and (b). Molecular analyses revealed a wild-type *KBTBD4* gene and a DNA methylation class distinct from PPTID-A and -B. Of note, most of the PPTID_wt_ in our cohort showed a large-cell morphology.


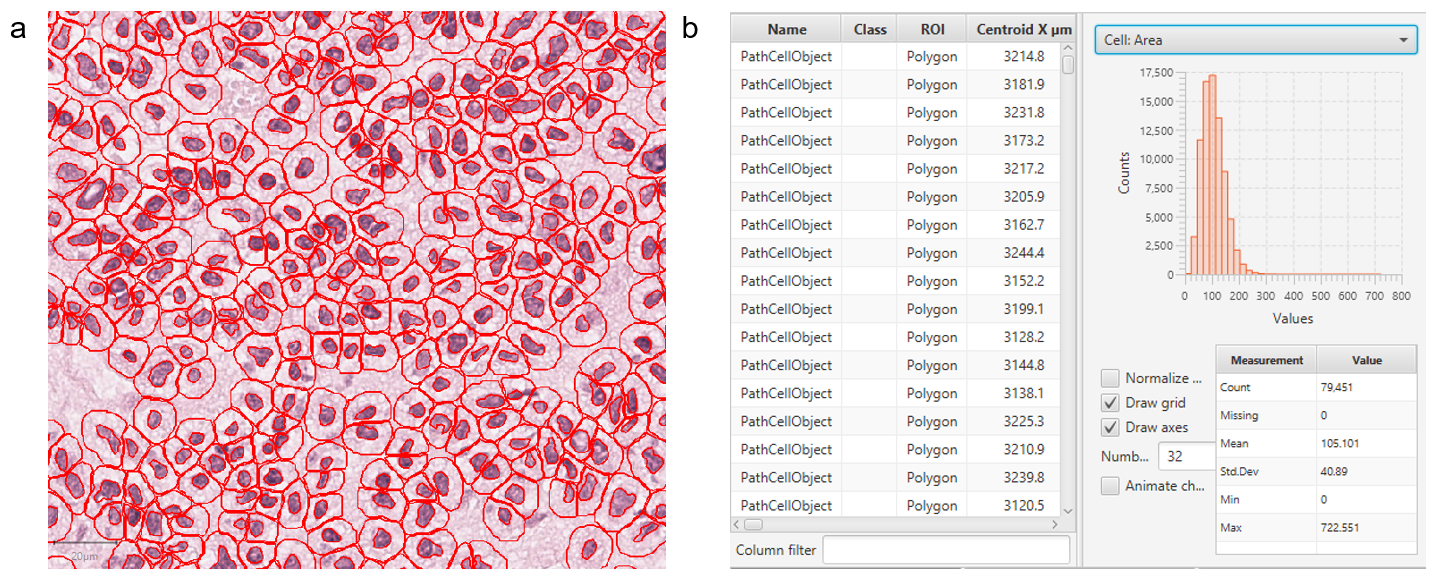


**Supp. Fig. 5**

Tumor cell area and count was measured by running Cell detection command in QuPath. In this case (H&E stain illustrated in Supp. Fig. 1), mean tumor cell area of 105 µm2 measured by QuPath and 79451 tumor cells were detected in an area of 12.3 mm2 equals a cell density of 6460 cells / 1mm2.


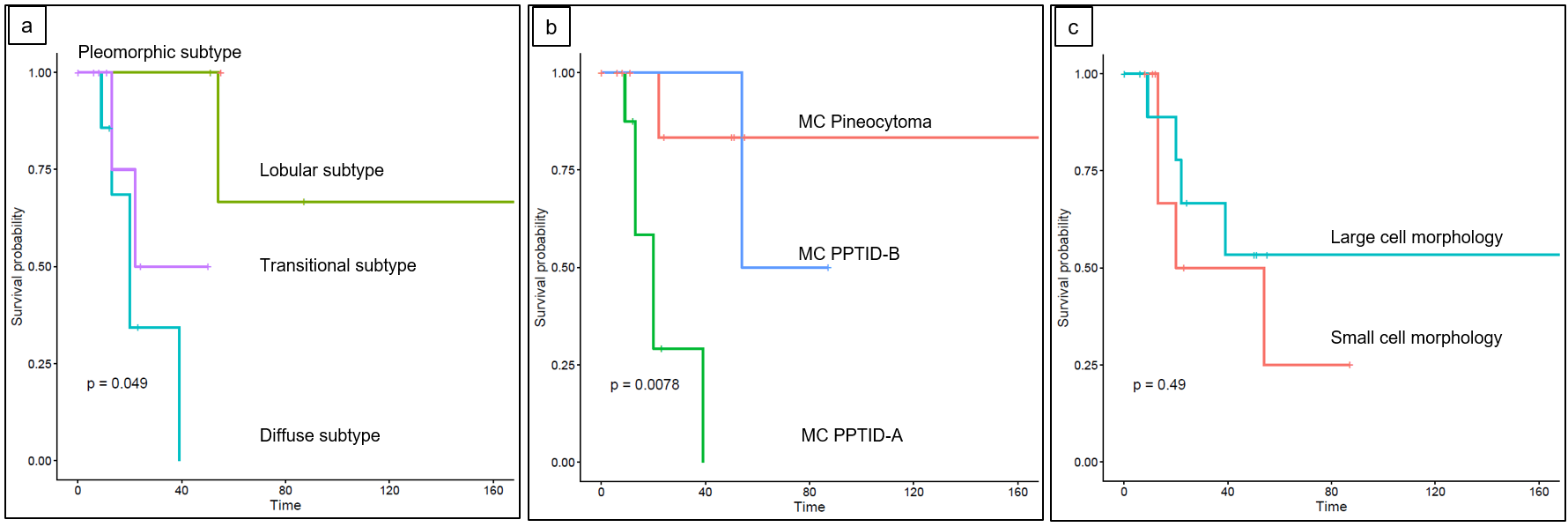


**
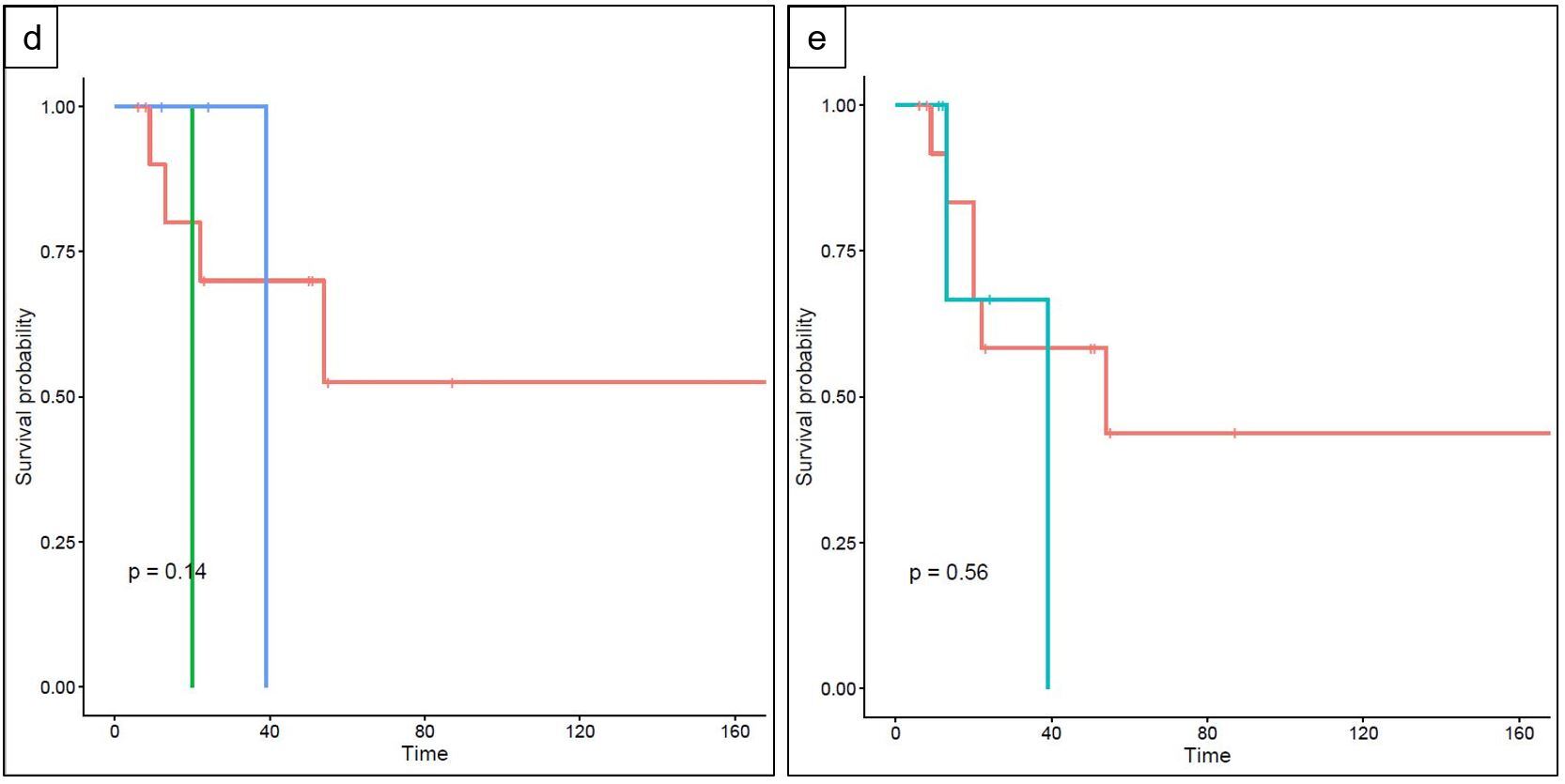
**

**Supp. Fig. 6**

Kaplan-Meier curves by Log-rank test. Diffuse morphological subtype (a, p-value 0.04), DNA methylation class of PPTID-A (b, p-value 0.007) were significantly associated with worse progression-free survival (PFS). The small cell versus large cell morphology (c, p-value 0.4), extent of resection (d, p-value 0.14) and adjuvant therapy (c, p-value 0.5) failed to correlate with the PFS.

| Identifier | Total | PPTID_wt_ | PPTID_mut_ |
| --- | --- | --- | --- |
| Age at diagnosis (mean) | 42 | 44 | 41 |
| Sex (F:M) | 10:9 | 5:4 | 5:5 |
| Recurrence | 8 | 1 | 7 |
| Resection    GTR  STR  Biopsy  N/A | 19  12  2  3  2 | 9  7  0  1  1 | 10  5  2  2  1 |
| Post-op Radiotherapy    No RT  Cranial only  Craniospinal | 14  3  2 | 7  2  0 | 7  1  2 |
| Mortality | 3 | 1 | 2 |

**Table 1 Summary of the clinical findings of patients with available survivial data.**

Follow-up data were available for 19 PPTIDs, with a mean follow-up period of 36.1 months (range: 6-169 months). Radiological tumor recurrence was evident in one PPTID_wt_ (out of 9) and seven PPTID_mut_ (out of 10) cases. Gross total resection (GTR), defined as the removal of more than 95 percent of the gadolinium-enhancing area in postoperative MRI, was achieved in seven cases of PPTID_wt_ and five cases of PPTID_mut_, respectively. No patient received chemotherapy. Among the patients who underwent diagnostic biopsy (no debulking), which included 2 PPTID_mut_ and 1 PPTID_wt_, all received postoperative radiotherapy; one PPTID_mut_ showed recurrence in MRI study 39 months after the biopsy. For PPTID_mut_ patients who underwent subtotal resection (STR) (n=2), no adjuvant therapy was administered following surgery, and both showed recurrences in MRI studies around 20 months after surgery. PPTID_mut_ patients who underwent GTR (n=5) were all followed up without postoperative adjuvant therapy, and three of them showed recurrence after 9, 13, and 54 months. Spinal metastasis was documented in two cases of PPTID_mut_, one case occurring together with local recurrence after 20 months and the other case occurring 6.4 years after the operation and 13 months after the local recurrence. Three cases resulted in death; one PPTID_wt_ after 45 months and two PPTID_mut_ after 101 and 69 months. GTR gross total resection, STR subtotal resection, N/A not available, RT radiotherapy, F female, M male

| Identifier | Nr. of cases;  Total (available follow up) | Hotspot Ki67 | NFP Score  (percent of cases scored 1) | Mitotic count (in 10 HPFs: 2.4 mm^2^) | Tumor cell area (mean in µm^2^) | Small  cell morphology  (mean cell area < 85µm^2^) | Tumor cell density (mean cell count/ 1mm^2^) | 13q loss |
| --- | --- | --- | --- | --- | --- | --- | --- | --- |
| PPTID_mut_ | 24 (10) | 8.4  (n=10) | 70%  (n=10) | 2.8  (n=24) | 78  (n=24) | 80%  (n=24) | 12160  (n=24) | 12%  (n=24) |
| PPTID_wt_ | 10 (9) | 6.1  (n=9) | 20%  (n=9) | 0.8  (n=10) | 100  (n=10) | 20%  (n=10) | 7954  (n=10) | 60%  (n=10) |

**Table 2 Summary of the histological and molecular findings.**
