# Supplementary material for: Genetical and epigenetical profiling identifies two subgroups of pineal parenchymal tumors of intermediate differentiation (PPTID) with distinct molecular, histological and clinical characteristics
Source: Acta Neuropathol. 2023 Sep 30;146(6):853–6. doi: 10.1007/s00401-023-02638-1 (PMC10627898; doi:10.1007/s00401-023-02638-1)
Supplement: Supplementary file 2 — Supplementary file2 (DOCX 22 KB) [file 401_2023_2638_MOESM2_ESM.docx]

**Supplementary Appendix**

Supplement to: Rahmanzade R, Pfaff E, et al. **Genetical and epigenetical profiling identifies two subgroups of Pineal parenchymal tumors of intermediate differentiation (PPTID) with distinct molecular, histological and clinical characteristics**

Corresponding authors:

**Felix Sahm, MD**

Department of Neuropathology

University Hospital Heidelberg

and

Clinical Cooperation Unit Neuropathology (B300)

German Cancer Research Center (DKFZ)

Im Neuenheimer Feld 224

69120 Heidelberg, Germany

Fon: +49-6221 56-37886; Fax: +49-6221 56-4566

[Felix.sahm@med.uni-heidelberg.de](mailto:Felix.sahm@med.uni-heidelberg.de)

**Material & Methods**

**Sample selection and clinical data**

The histological slides and molecular findings of 110 pineal cases from our internal databank with available diagnostic slides and DNA material have been reviewed. Accordingly, the cases were classified as follows: 34 PPTIDs, 20 PPTR, 36 PB, 8 pineocytoma, 2 pineal cyst, 10 normal pineal gland.

The following demographical and clinical data were gathered for a subset of cases with available follow-up data (n=19): age at diagnosis, sex, extend of resection (gross total resection, subtotal resection, diagnostic biopsy), date of operation, date of the last radiological examination, death status, radiological evidence of recurrence or neuroaxis spreading, treatment modality (none, radiotherapy, chemotherapy).

**Histology and immunohistochemistry**

All cases were subject to the histological review based on the most recent WHO classification of brain tumors [1]. Histological features such as growth pattern, tumor cell size (small cell versus large cell morphology), tumor cell density, necrosis and mitotic counts, shown previously to have prognostic significance, were assessed. Tumors with available paraffin blocks were subjected to immunohistochemical workup (n=19). H&E staining was performed according to standard protocols. Immunohistochemistry was carried out on a Ventana BenchMark ULTRA Immunostainer (Ventana Medical Systems, Tucson, AZ, USA). Following Antibodies were used: Ki-67 (mouse monoclonal, clone MIB-1, dilution 1:100), Synaptophysin (rabbit monoclonal, clone MRQ-40, dilution 1:50, Merck), Neurofilament Protein (mouse monoclonal, clone 2F11, dilution 1:200). For Ki-67 the Ventana OptiView DAB IHC Detection Kit (Ventana Medical Systems) were used. Slides were scanned with an Aperio AT2 scanner and visualized with Aperio Image Scope v12.4.3.7001 (Aperio, Leica Biosystems, Deer Park, IL, USA). QuPath (version 0.2.0.), an open source image analysis software, was used to measure tumor cell area, tumor cell density and hotspot Ki-67 (Fig. 1 and supplementary Fig. 6), as described before (Paik S. et al; NPJ Breast Cancer. 2021 Feb). Hotspot Ki-67 was measured for a subset of PPTIDs with available survival data (n=19). To increase the specificity and reproducibility of Ki-67 positive cell detection, we applied the following modifications: (1) the cut-off value of staining intensity has been set 0.5 (default value of 0.2), (2) analysis conducted with a grid size of 500µm x 500µm, and (3) grids with less than 500 cells were excluded (supplementary Fig. 1). Neurofilament expression has been assessed as proposed before [4-6]. Tumors showing no immunoreactivity against neurofilament or staining in less than 10% of tumour cells have been scored 1 and the rest showing a moderate to high immunolabelling have been scored 0, as proposed before [4,6]. Hotspot Ki67 was scored based on the median of the values as 0 (Hotspot Ki67 less than 8%) and 1 (higher than 8%).

**DNA methylation profiling and copy number analysis**

DNA was extracted from formalin-fixed paraffin-embedded (FFPE) samples at Department of Neuropathology Heidelberg. Genome-wide DNA methylation data was obtained using the Infinium Methylation EPIC (850k) or Infinium HumanMethylation450 (450k) BeadChip array (Illumina, San Diego, CA, USA), according to the manufacturer’s instructions and as previously described. Copy number profiles were generated from 450k and EPIC methylation array data using the conumee Bioconductor package version 1.12.0, as described before [2]. All computational analyses were performed in R version 3.6.0 (R Development Core Team 2016, [https://www.R-project.org](https://www.r-project.org/)). Copy-number profiles were visually inspected for evaluation of chromosomal gains and losses. Copy-number variation (CNV) load has been calculated using a local software.

**Next-generation DNA sequencing**

A total of 37 cases including 34 PPTIDs and 3 pineocytoma were subjected to DNA-sequencing. The Next-generation sequencing was performed at the Department of Neuropathology Heidelberg using a capture-based next-generation DNA sequencing approach on a NextSeq 500 or NovaSeq 6000 instrument (Illumina) applying a custom brain tumor panel as previously described before (Sahm F, et al; Acta Neuropathol. 2016 Jun). *KBTBD4*, the Kelch repeat- and BTB domain-containing protein 4, as the gene of interest was further investigated using the Integrative Genomic Viewer (IGV) for the presence of small insertions. The *KBTBD4*-status was scored 1 in the case that insertions were detected and 0 for wild-type gene.
